# Inborn errors of metabolism and the human interactome: a systems medicine approach

**DOI:** 10.1007/s10545-018-0140-0

**Published:** 2018-02-05

**Authors:** Mathias Woidy, Ania C. Muntau, Søren W. Gersting

**Affiliations:** 1 University Children’s Hospital, University Medical Center Hamburg Eppendorf, Martinistrasse 52, 20246 Hamburg, Germany; 2 Department of Molecular Pediatrics, Dr. von Hauner Children’s Hospital, Ludwig-Maximilians-University Munich, Lindwurmstrasse 4, 80336 Munich, Germany

**Keywords:** Inborn errors of metabolism, Interactome, Network medicine, Disease module, Protein-protein interaction

## Abstract

The group of inborn errors of metabolism (IEM) displays a marked heterogeneity and IEM can affect virtually all functions and organs of the human organism; however, IEM share that their associated proteins function in metabolism. Most proteins carry out cellular functions by interacting with other proteins, and thus are organized in biological networks. Therefore, diseases are rarely the consequence of single gene mutations but of the perturbations caused in the related cellular network. Systematic approaches that integrate multi-omics and database information into biological networks have successfully expanded our knowledge of complex disorders but network-based strategies have been rarely applied to study IEM. We analyzed IEM on a proteome scale and found that IEM-associated proteins are organized as a network of linked modules within the human interactome of protein interactions, the IEM interactome. Certain IEM disease groups formed self-contained disease modules, which were highly interlinked. On the other hand, we observed disease modules consisting of proteins from many different disease groups in the IEM interactome. Moreover, we explored the overlap between IEM and non-IEM disease genes and applied network medicine approaches to investigate shared biological pathways, clinical signs and symptoms, and links to drug targets. The provided resources may help to elucidate the molecular mechanisms underlying new IEM, to uncover the significance of disease-associated mutations, to identify new biomarkers, and to develop novel therapeutic strategies.

**Electronic supplementary material:**

The online version of this article (10.1007/s10545-018-0140-0) contains supplementary material, which is available to authorized users.

## Introduction

The human genome consists of ~25,000 protein coding genes and most proteins carry out cellular functions by interacting with other small molecules or macromolecules. In the network resulting from all these interactions—the human interactome—the cellular components, such as proteins, RNA, or metabolites, serve as nodes and their interactions are commonly depicted as edges (Schadt 2009; Barabási et al 2011; Caldera et al 2017). Inborn errors of metabolism (IEM) are the consequence of genetic variation, but the disease phenotype does not only result from functional alteration of the affected gene product, perturbations rather spread along the links of the underpinning cellular networks (Schadt 2009; Barabási et al 2011; Menche et al 2015; Piñero et al 2016; Caldera et al 2017).

Proteins are the drivers of cellular function and the entirety of protein-protein interactions forms a large sub-network within the human interactome (Vidal et al 2011). A complete high-quality map of the protein interaction network is of fundamental importance for the understanding of diseases (Vidal et al 2011; Luck et al 2017). The analysis of these networks with the emerging tools of network medicine may help to understand the cellular mechanisms underlying IEM, the relationships between different IEM or between IEM and other diseases.

Biological networks are not random but governed by organizing principles. They are scale-free, *i.e.*, there are many nodes with few neighbors and few nodes with many neighbors, which are called hubs (Barabási et al 2011; Pavlopoulos et al 2011). Disease-associated proteins are not scattered randomly in the interactome, but tend to interact with each other (Goh et al 2007; Feldman et al 2008; Caldera et al 2017). A cluster of disease-associated proteins in the same network neighborhood forms a subgraph, which constitutes a disease module (Barabási et al 2011; Menche et al 2015). These modules may be tissue-specific (Kitsak et al 2016) and personalized gene expression pools that relate to these modules impact individual disease expression (Menche et al 2017). Within a disease module, different diseases may arise from common mechanisms and display overlapping phenotypes as shown for complex diseases, such as inflammation, asthma, and cardiovascular diseases (Menche et al 2015; Sharma et al 2015; Ghiassian et al 2016).

However, network medicine approaches have not been used to systematically study IEM (Argmann et al 2016) as a heterogeneous group of disorders affecting virtually all human biochemical pathways and impairing the function of many organs. A hierarchical classification of IEM provided by the Society for the Study of Inborn Errors of Metabolism (SSIEM) lists 612 diseases with MIM numbers (www.omim.org) in 15 disease groups (www.ssiem.org/resources/IEC.asp). By using the emerging tools of network medicine, we aimed to investigate whether the entity of IEM is indeed organized as disease modules in the interactome of protein interactions and how these relate to each other. We approached IEM on a proteome scale and established an IEM-specific interactome (IEMi), which comprised 298 of 427 IEM-related proteins. To further improve the clinical relevance of the strategy we investigated relations of the IEMi to other non-IEM diseases, integrated database information such as functional annotations from the gene ontology, phenotypic features, and links to drugs or biologically active compounds.

## Results

### The IEM interactome

**Fig. 1:**
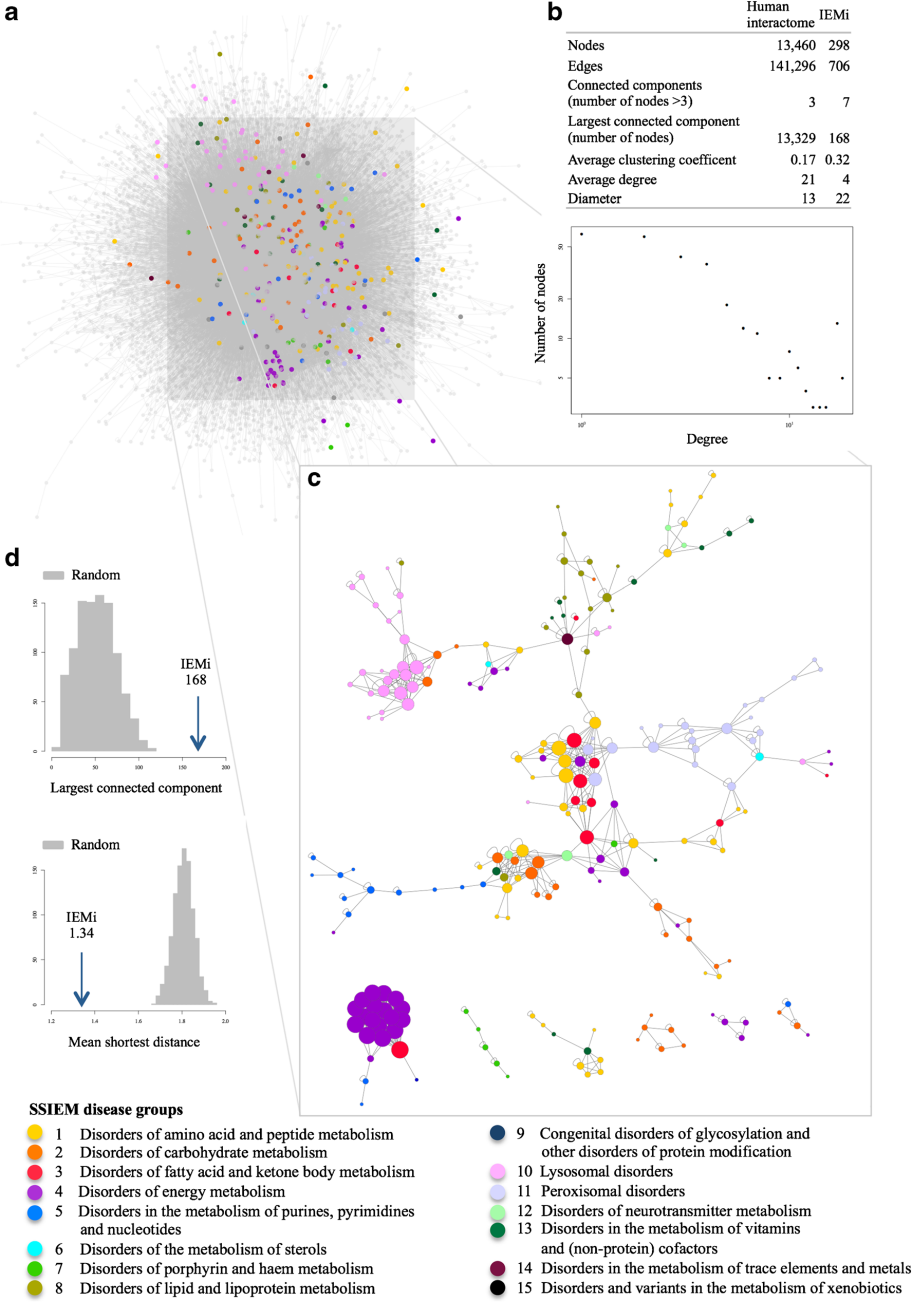
From the human interactome to the IEM interactome. **a** Network representation of the interactome of all human protein-protein interactions. This interactome represents a complete map of all known interactions between proteins and is a part of the full human interactome including interactions between proteins and DNA, RNA or metabolites. Nodes are proteins and they are connected by an edge when two proteins interact. Colored nodes are associated with an inborn error of metabolism (IEM) and colors depict different IEM disease groups. IEM-associated proteins that interact with other IEM-associated proteins define the IEM interactome (IEMi) within the human interactome. **b** Network parameters of the complete human interactome and the IEMi. Log-log plot of the degree distribution for all nodes of the IEMi against the number of nodes points to a scale-free network. **c** The inset shows the seven largest connected components (*n* > 3 nodes) of the IEMi. The size of a node is proportional to the number of its neighbors, which are interaction partners (the number of neighbors defines the degree of a node within a network). For a detailed scalable view including protein names, see Suppl. Fig. 1. **d** The distribution of largest connected component sizes and mean shortest distances for randomly chosen sets of proteins from the human interactome compared to the IEMi (arrows). The number of randomly chosen proteins is similar to that of the IEMi. The arrows show the observed values for largest connected component (*z*-score, 5.32) and mean shortest distances (*z*-score, −10.09) of the IEMi, which are both significantly different from random expectation

As a platform for network medicine analyses, Menche et al (2015) compiled a human interactome (Fig. 1a) that consists of 13,460 proteins connected by 141,296 high-confident interactions including binary protein-protein interactions, regulatory interactions and metabolic pathway interactions (Fig. 1b). Following the SSIEM classification, 427 disease genes are known to be associated with an IEM (Suppl. Table 1) and 376 of their gene products, the IEM-associated proteins, were identified in the human interactome. Within the set of 376 IEM-associated proteins, 298 proteins established 706 interactions with each other, resulting in the IEM interactome (IEMi) (Fig. 1c, Suppl. Fig. 1). The IEMi comprised seven connected components of more than three nodes, where the largest connected component contained 168 IEM disease proteins linked to 14 out of 15 IEM disease groups. The second largest connected component with 22 nodes mainly consisted of proteins associated with disorders of energy metabolism (group 4), while the nine proteins that formed the third largest connected component are associated with disorders of amino acid and peptide metabolism (group 1) and disorders in the metabolism of vitamins and (non-protein) cofactors (group 13).

The *degree* of a node within a network describes the number of connections to other nodes, in the case of a protein interaction network the number of interactions a given protein establishes with other proteins. As previously shown for other biological networks (Barabási et al 2011), the *degree* distribution of the IEMi followed a power law, indicating a scale-free biological network with many proteins with low *degree* and few proteins with high *degree* (Fig. 1b). The analysis of the IEMi showed that, on average, IEM-associated proteins have only four interaction partners, whereas the average *degree* of the human interactome is 21. Highly connected nodes, the hub proteins, take over specific biological roles and can be subdivided by their dynamical behavior in networks (Pavlopoulos et al 2011; Seebacher and Gavin 2011). They can either constitute a central node within a module or connect several subgraphs within a network. Within the IEMi we identified 45 nodes with a degree ≥10. The *clustering coefficient* of a node describes the tendency with which the neighbors of this node also interact with each other. The average *clustering coefficient* (cl) of the IEMi was 0.32; thus, larger than for the human interactome (cl, 0.17). The *diameter* is the largest distance between any two pairs of nodes in a network. The *diameter* was 22 for the IEMi and 13 for the human interactome. The *largest connected component* of the IEMi (168, *z*-score 5.3) was larger than the random expectation (52.4), whereas the network-based *mean shortest distance* of IEM-associated proteins was smaller (1.34, *z-score* − 10.1) than the random expectation (1.81) (Fig. 1d). Taken together, the results from the analysis of the network-based measures *largest connected component* and *mean shortest distance* support the notion that IEM-associated proteins tend to locate to the same network neighborhood in the human interactome. In addition, we observed two different patterns of clustering within the IEMi. Proteins associated with certain disease groups built rather homogenous clusters and consequently were separated from other IEM-associated proteins (lysosomal disorders; peroxisomal disorders; disorders in the metabolism of purines, pyrimidines and nucleotides; disorders in the metabolism of lipids and lipoproteins; disorders of energy metabolism). Proteins associated with other disease groups either contributed to heterogeneous clusters central to the largest connected component or they were distributed over remote areas of the network.

### IEM disease modules

**Fig. 2:**
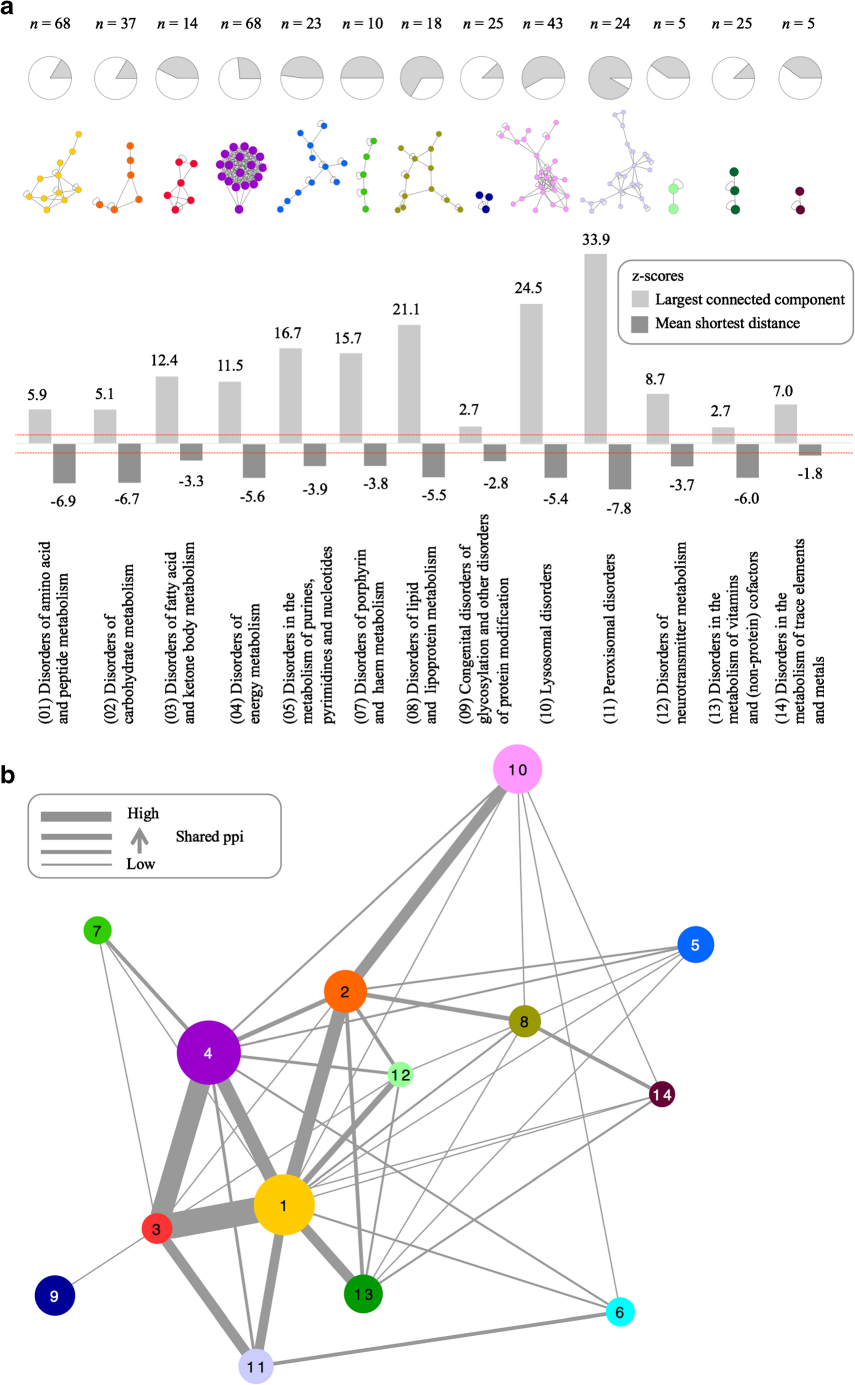
IEM disease modules. **a** Identification of disease modules within IEMi that are specific for IEM disease groups. The *z*-scores for the observed values of the largest connected components (light gray) and mean shortest distances (dark gray) are shown. The red lines indicate *z*-scores of +1.6 and −1.6 as thresholds for the level of significance (*p*-value <0.05). The pie charts give the share of proteins involved in the largest connected component (gray area) compared to the number of proteins associated with the respective IEM disease group. Disease groups 6 (disorders of the metabolism of sterols) and 15 (disorders in the metabolism of xenobiotics) were excluded from the analysis due to low numbers of associated proteins or lack of interactions. **b** IEMi disease group network. Nodes represent IEM disease groups. The thickness of edges is proportional to the number of shared interactions. The node size corresponds to the number of proteins associated with the respective IEM disease group

To test the disease modules hypothesis for each of the IEM disease groups following the SSIEM classification, we calculated the network-based measures *largest connected component* and *mean shortest distances* for all disease groups (Fig. 2a). The analysis was performed for all IEM-associated proteins mapping to the human interactome (*n*, 376). Disease groups 6 (disorders of the metabolism of sterols) and 15 (disorders in the metabolism of xenobiotics) were excluded from the analysis due to low numbers of associated proteins or lack of interactions. The IEM disease groups were compared to sets of nodes with the respective numbers of nodes that represented the general characteristics of the human interactome. We computed *z*-scores for each comparison and observed that 13 disease groups established significant disease modules (*largest connected component*, *z*-scores >1.6; *mean shortest distance*, *z*-scores <1.6). The largest disease modules were identified for lysosomal disorders (25 nodes), peroxisomal disorders (22 nodes), and disorders of energy metabolism (18 nodes), where the latter formed a highly interlinked motif. Three medium-sized modules arising from the disease groups 1, 5, and 8 were less interlinked. The remaining modules either were not interlinked (disease group 7) or were very small (disease groups 12–14).

Next, we aimed to investigate the overlap between the IEM disease groups based on protein interactions (Fig. 2b) and found 211 interactions between proteins belonging to different disease groups. Disorders of amino acid and peptide metabolism (disease group 1) had a central position within this network. This group formed a subnetwork together with disorders of carbohydrate metabolism (group 2), disorders of fatty acid and ketone body metabolism (group 3), and disorders of energy metabolism (group 4) that accounted for 44% of all interactions between the disease groups. In addition, lysosomal disorders (group 10) shared 6% of interactions with disorders of carbohydrate metabolism (group 2). Peroxisomal disorders (group 11) showed significant overlap with disorders of amino acid and peptide metabolism (group 1) as well as disorders of fatty acid carbohydrate metabolism (group 3). Congenital disorders of glycosylation and other disorders of protein modification (group 9) were, except for one interaction to disorders of fatty acid and ketone body metabolism (group 3), disconnected from the other IEM disease groups.

Disease groups with a high degree of self-organization, *i.e.*, many nodes belonging to the same disease group are organized within the specific module (groups 5, 7, 8, 10, 11), established few interactions to other disease groups. In the case of disease groups showing a low degree of self-organization, *i*.*e*., only a small share of the nodes is organized within the specific module, the degree of interconnection with other disease groups increased (groups 1, 2, 4). Taken together, we identified disease groups with a high degree of interaction-mediated overlap to other IEM, whereas others constituted more distinct entities based on their interaction behavior. Disease group 3 (disorders of fatty acid and ketone body metabolism) represented a special case. Here we observed a compact specific disease module where half of the proteins belonging to the disease group contributed to the module. In addition to this rather high degree of self-organization strong interactions were established with disease groups 1 (disorders of amino acid and peptide metabolism), 4 (disorders of energy metabolism), and 11 (peroxisomal disorders) and weak interactions were established with disease groups 2, 7, 9, and 12.

### Relationship between IEM and non-IEM pathways and diseases

**Fig. 3:**
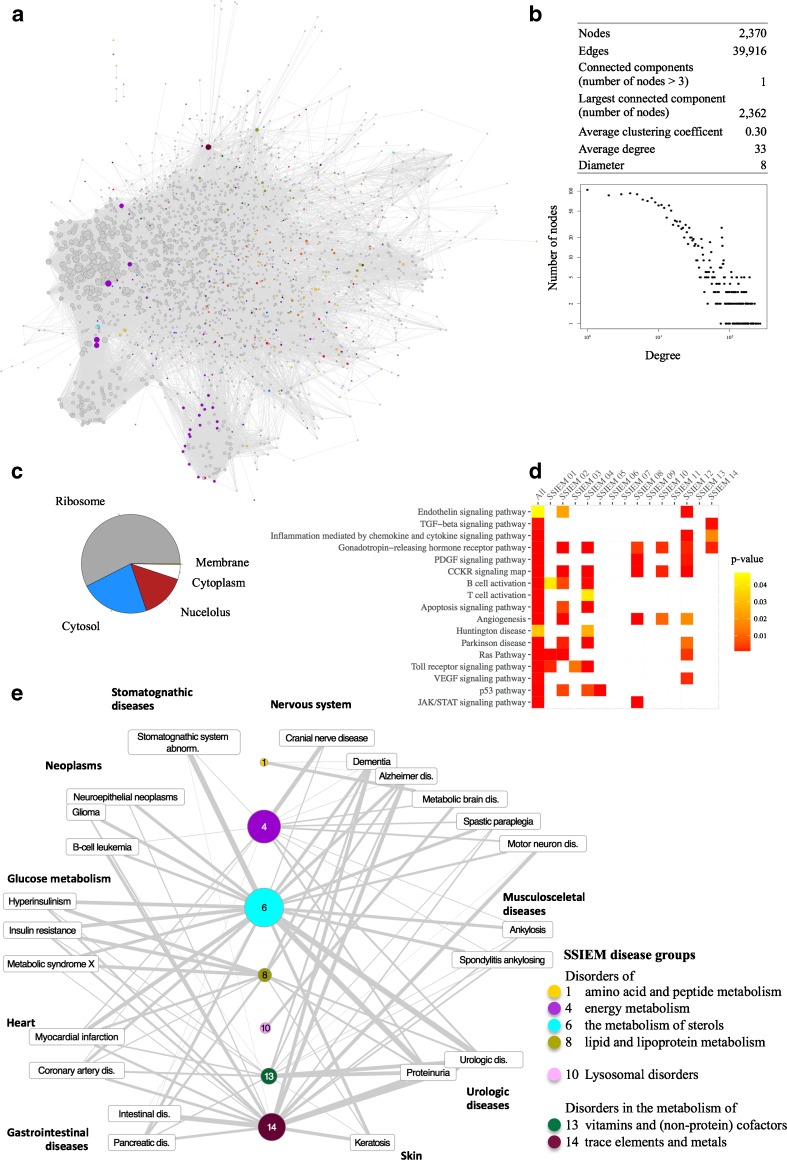
The expanded IEM interactome. **a** Expanded IEM interactome (eIEMi). The IEMi was expanded to include first-order interactions of IEM-associated proteins (colored nodes) to non-IEM proteins (gray nodes). The size of a node corresponds to the number of its neighbors (degree). **b** Network parameters of the eIEMi; a log-log plot of the degree distribution of all nodes of the eIMEi against the number of nodes points to a scale-free network. **c** Pie chart of the calculated fold enrichment of cellular compartment distribution for proteins with degree >100 of the eIEMi. Ribosomal, cytosolic, and nuclear proteins are overrepresented. Membrane proteins are underrepresented. **d** Heat map of enriched PANTHER pathways of non-IEM proteins interacting with proteins of the different IEM disease groups. Cells are colored according to their respective p-value. Metabolic pathways were excluded from this matrix representation. **e** The eIMEi disease network reveals associations of IEM disease groups with non-IEM diseases based on protein interactions. Rectangle nodes are MeSH terms of non-IEM diseases. Colored nodes represent IEM disease groups, the size is proportional to the shared links with general disease groups. The width of an edge is proportional to the network-based relationship, *i.e.*, the edge width negatively correlates with a calculated separation score

To uncover disease-disease relationships of IEM with other non-IEM disease classes we sought to expand the IEMi. We pursued the hypothesis that the location of IEM disease modules in the neighborhood to other disease genes in the human interactome implies shared biological pathways, similar clinical signs and symptoms, and common disease mechanisms. We extracted binary interactions of IEM-associated proteins with non-IEM proteins in the human interactome, retrieved 1994 first order interaction partners and additionally included interactions within this set of proteins (Suppl. Table 2). This resulted in an expanded IEM interactome (eIEMi) of 2370 proteins linked by 39,916 interactions (Fig. 3a). The eIEMi consisted of one connected component of more than three nodes (*largest connected component*, 2362), the average *degree* increased to 33 as compared to 4 for the IEMi and the *diameter* of the eIEMi decreased to 8 (Fig. 3b). Again, the degree distribution followed a power law, indicating a scale-free biological network with few highly connected hub proteins. We characterized eIMEi hub proteins with a *degree* > 100, which are important for the topology and function of the network, with respect to their cellular distribution and observed overrepresentation of proteins located in the ribosome (fold enrichment (FE), 48.01; *p*-value, 1.81E-90), cytosol (FE, 18.92; *p*-value, 1.13E-66), nucleolus (FE, 12.34; p-value, 1.18E-12), and cytoplasm (FE, 4.05; *p*-value, 3.36E-33). Membrane proteins were underrepresented (FE, 0.2; *p*-value, 0.0029) (Fig. 3c).

Next, we performed a pathway enrichment analysis for pathways associated with non-IEM proteins in the eIEMi. Besides enrichment for metabolic pathways and immune signaling pathways, we found significant enrichments for angiogenesis (FE, 2.97; p-value, 5.51E-8), apoptosis signaling pathway (FE, 3.86; p-value, 2.76E-10), and for the two diseases Parkinson disease (FE, 3.44; p-value, 1.95E-6) and Huntington disease (FE, 2.18; p-value, 0.034) (Fig. 3d and Suppl. Table 3). The gonadotropin releasing hormone receptor pathway, the CCKR signaling pathway, and angiogenesis were associated with ≥4 IEM disease groups. A high number of different pathways was enriched for non-IEM proteins interacting with IEM-associated proteins linked to disorders of carbohydrate metabolism, energy metabolism or neurotransmitter metabolism (groups 2, 4, 12), whereas no pathway enrichments were observed for non-IEM proteins associated with disease groups 6, 7, 9, 11, 13.

In order to evaluate the overlap of IEM disease groups with non-IEM diseases, we analyzed their network-based distances using a separation score, which is a network-based measure for pathobiological and clinical similarity (Menche et al 2015; Caldera et al 2017), and identified the related phenotypic features (MeSH terms). We calculated separation scores for IEM disease groups 1–14 against a set of 299 non-IEM diseases and ranked separation scores for each of the 4186 resulting pairs (Fig. 3e and Suppl. Table 4). Disorders of energy metabolism and metabolism of sterols (groups 4, 6) shared the most pronounced overlap to other diseases, reflected by the highest number of established links. The closest similarities among diseases, reflected by low separation scores, were observed between disorders of sterols metabolism and urologic diseases (−0.080), stomatognathic system abnormalities (−0.054), and chronic B-cell leukemia (0.054), respectively. Disorders in the metabolism of trace elements and metals were closely linked to intestinal (0.003) as well as urologic diseases (0.013). Additionally, disorders of energy metabolism were strongly connected to cranial nerve diseases (0.066). Dementia, Alzheimer disease (AD), and motor neuron diseases displayed similarities with IEM disease groups 6, 8, 13, and 14. In particular, we found associations of disorders of metabolism of sterols (group 6) with AD.

The association between serum cholesterol levels and cerebral amyloidosis in the pathogenesis of AD (Puglielli et al 2003; Vaya and Schipper 2007; Reed et al 2014) is not likely due to similar genetic predispositions (Proitsi et al 2014). However, there is evidence for a mechanistic link between β-amyloid protein toxicity and mitochondrial cholesterol trafficking (Barbero-Camps et al 2014), and the HMG-CoA-reductase inhibitor Simvastatin has been demonstrated to affect β-amyloid peptides (Zandl-Lang et al 2017). In addition, we found associations of disorders in the metabolism of trace elements and metals (group 14) with AD (Fig 3e). Recently, polymorphisms in the ATP7B gene, which is responsible for Wilson disease and a part of IEM-disease group 14, have been associated with increased risk of AD (Mercer et al 2017). These findings underlined how the identification of such network-based connections and the transfer of knowledge between associated disease groups may help to elucidate molecular mechanisms of disease and to elaborate new therapeutic strategies.

### IEM drug–target network

**Fig. 4:**
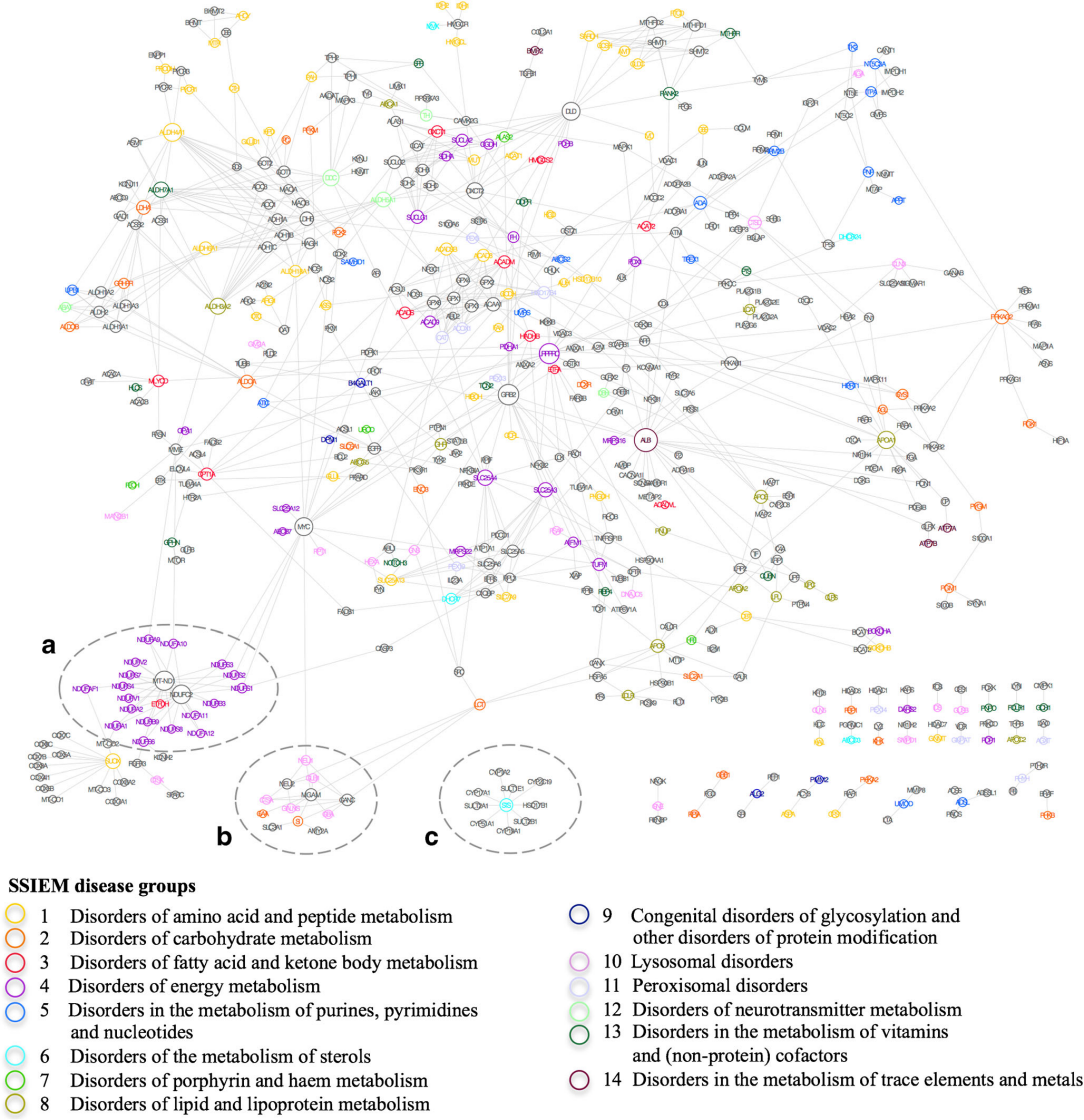
The eIEMi drug target network. The eIEMi drug target network shows interactions of IEM-associated proteins with non-IEM proteins, which are listed as drug targets in the drug bank (www.drugbank.ca). For all proteins, gene names are given as short names (UniProt), nodes are colored according to the IEM disease group and gray colored nodes represent non-IEM proteins. The size of a node is proportional to the number of its neighbors (degree)

The observed similarities as to underlying biological pathways, disease mechanisms, and phenotypes between IEM-associated proteins and non-IEM proteins in the eIMEi network allow for the hypothesis of the effectiveness of common treatment strategies. Consequently, a drug may have impact on proteins that are linked to the drug target protein in a protein interaction network. We included drug-target information from the drugbank database (Law et al 2014) into the eIMEi, identified 634 approved drugs or active compounds that target 316 non-IEM proteins, and mapped their protein interactions with IEM-associated proteins in the eIEMi space. The resulting IEM drug-target network consisted of 538 proteins and 845 interactions (Fig. 4 and Suppl. Table 5). Besides a share of otherwise unlinked binary interactions between non-IEM drug targets and IEM-associated disease proteins, the IEM drug-target network showed a large connected component with 465 nodes. This sub-network was organized by several highly interlinked hub proteins, both IEM and non-IEM proteins. Hence, different groups of drug targets were interconnected by IEM-associated proteins or IEM disease modules were interconnected by non-IEM drug targets. To exemplify approaches toward the evaluation of therapeutic concepts and identification of novel treatment options for IEM, we discuss three scenarios. (A) An IEM disease module linked by non-IEM drug targets. The IEM disease module for disorders of energy metabolism (group 6) was interconnected by two hub proteins, which are subunits of the mitochondrial respiratory chain complex I, NADH-ubiquinone oxidoreductase (MT-ND1), and a NADH dehydrogenase (NDUFC2). MT-ND1 is listed in the drugbank database as target of volatile anesthetics, such as isoflurane and halotane, whereas NDUFC2 is an off-target of the β-blocker carvedilol. (B) Multiple links between IEM-associated proteins and non-IEM drug targets within a disease module. A disease module consisting of IEM-associated proteins linked to lysosomal disorders and disorders of carbohydrate metabolism contains different non-IEM drug targets. Here, intestinal maltase-glucoamylase (MGAM) and neutral alpha-glucosidase C (GANC) are targeted by alpha-glucosidase inhibitors. In addition, sialidase 1 is a target of low-molecular weight heparins and sialidase 2 of the neuramidase inhibitor oseltamivir. (C) An IEM-associated protein linked to multiple non-IEM drug targets. The steroid sulfatase A, which is deficient in X-linked ichthyosis, is central to a drug-target module addressed by imidazole or nicotinic acid derivatives (rifampicin, isoniazid, metronidazole).

Binary interactions of IEM-associated proteins and non-IEM drug targets may represent mechanistic links on the molecular level, and hence give rise to novel therapeutic approaches. The solute carrier family 25 member 4 gene (SLC25A4) encodes for adenine nucleotide translocase type 1 (ANT1) that interacts with mitochondrial peptidyl-prolyl cis-trans isomerase F (PPIF) in the IEM drug target network. Mutations in SLC25A4 are associated with different mitochondrial disorders including phenotypes of mitochondrial myopathy, exercise intolerance, cardiomyopathy, and progressive external ophtalmoplegia (Kaukonen et al 2000; Strauss et al 2013; Thompson et al 2016; Tosserams et al 2017). Cyclosporine A targets the mitochondrial permeability transition pore (mPTP) via the mPTP regulator PPIF and CypD-ANT1 and has been shown to improve mitochondrial function (Merlini et al 2008; Qiu et al 2014). In patients, cyclosporine A acts as a neuroprotective agent against stroke (Osman et al 2011) and is cardioprotective in events of ischemia (Hausenloy et al 2012). The interaction of SLC25A4 with PPIF in our drug-target network points to a potential therapeutic effect of cyclosporine A in the treatment of mitochondrial disorders linked to SLC25A4.

## Discussion

The group of IEM displays a marked heterogeneity and IEM can affect all functions and organs of the human organism. The currently most applied classification defines IEM disease groups according to three different criteria (i) the metabolites pertained by the genetic variation such as disorders of carbohydrate metabolism; (ii) the affected biological pathways such as disorders of glycosylation; (iii) the cellular localization of the IEM-associated proteins such as lysosomal disorders. A considerable number of IEM have been discovered over the past 100 years and particularly the more frequent among them are well studied. However, also due to the recent advances in genomics, many new, partly very rare IEM have been discovered, for which satisfying knowledge about diagnostics, care, and treatment is often not available.

Besides their heterogeneity, IEM share that their associated proteins function in metabolism. The latter is a huge functional unit that, as all extended functional pathways, is organized in biological networks. Network-based studies gave rise to the disease module hypothesis (Barabási et al 2011; Vidal et al 2011) where diseases with an overlap in biological networks show significant symptom similarity and common disease mechanisms, whereas diseases residing in separated network neighborhoods are phenotypically distinct. In a systematic investigation to uncover relationships between human diseases, Menche et al identified peroxisomal disorders as a distinct disease module in the human interactome (Menche et al 2015). Systematic approaches toward human disease that integrate database information and -omics data into biological networks have proven useful to further our understanding of common or complex disorders (Menche et al 2015; Sharma et al 2015; Ghiassian et al 2016). However, network-based strategies have been rarely applied to study the complex nature of IEM (Argmann et al 2016). Our study aimed to evaluate the expediency of such approaches using the human interactome and other existing biological or disease-associated datasets.

We used publicly available protein-protein interaction data between IEM-associated proteins and showed that IEM-associated proteins tend to locate to the same neighborhood within the human interactome. We termed the resulting sub-network IEM interactome (IEMi). The identification of IEM-specific disease modules revealed that the IEMi indeed contained allocations that correspond to the IEM-disease groups. Certain disease modules were highly interlinked to each other, on the other hand, we identified disease modules in the IEMi that consisted of proteins from many different disease groups. Notably, some IEM-associated proteins were not part of the IEMi and based on our network medicine approach did not show any overlap with other IEM. Therefore, the IEMi may offer a platform to systematically explore not only the molecular complexity of a particular disorder, but also the relationships among apparently distinct pathophenotypes of different IEM disease groups. In our study, we used a high-quality and comprehensive human interactome that has been curated recently (Menche et al 2015). However, it is estimated that current high-throughput methods cover less than 20% of all potential pairwise interactions in the human cell (Caldera et al 2017*)*. Therefore, the lack of associations between certain IEM-disease groups has to be evaluated carefully considering this incompleteness.

The central position of metabolism in the functional network of human biology implies a plethora of links that IEM-associated proteins can establish with non-IEM proteins including related cellular pathways and pathomechanisms in human disease. The expanded IEMi (eIEMi) explored the overlap between IEM and the human interactome through integration of database information on biological pathways, phenotypic features, and drugs or other active compounds. The graphical workup provided in this study exemplified network medicine approaches for IEM, offered resources in the supplementary material, and may be useful for the investigation of shared molecular functions, disease mechanisms, and the evaluation of existing or novel therapeutic concepts for IEM.

The transfer of knowledge within single disease groups, between related IEM disease modules or between IEM and non-IEM diseases will help to elucidate the molecular consequences associated with new disease genes, to uncover the significance of disease-associated mutations, to identify new biomarkers, and to expand the druggable space.

## Materials and methods

### Curation of disease genes associated with IEM

A classification of IEM is provided by the SSIEM. We downloaded the classification file at http://www.ssiem.org/resources/IEC.asp on March 15, 2015. Mapping of IEM diseases to associated disease genes was done in a web-based manner using the OMIM API (https://www.omim.org/help/api). This resulted in 427 IEM-related disease genes each assigned to the respective SSIEM disease group (Suppl. Table 1). We found only one disease protein associated with SSIEM class 15.

### Constructing the IEM interactome (IEMi) and the expanded IEM interactome (eIEMi)

We used protein-protein interaction data from a previously curated high-quality interactome (Menche et al 2015). In order to map protein-protein interaction data to the curated IEM-related proteins we used Entrez Gene ID retrieved from the HGNC database (http://www.genenames.org, downloaded Feb 5, 2017). For the construction of the IEMi, we extracted only protein interactions that directly linked IEM-related proteins resulting in 708 IEM-to-IEM protein interactions between 298 different proteins. To expand the IEMi we included protein interactions of first order proteins. These are non-IEM proteins that directly interact with IEM-associated disease proteins. The eIEMi contains IEM-to-IEM, protein interactions to first order proteins and protein interactions between first order proteins, in total 39,916 links. The 1994 first order proteins are listed in Suppl. Table 2.

### Calculation of *z*-score

Z-scores for the observed values V of largest connected component size and mean shortest distances were calculated as follows:

$$ z- score=\frac{V- mean\left({V}_{Random}\right)}{stand\left({V}_{Random}\right)}, $$

where mean(V_Random_) and stand(V_Random_) indicate the mean value and standard deviation of the random expectation.

### Enrichment analysis

Enrichment analysis was performed using the web-interface provided by PANTHER (http://pantherdb.org, Panther Annotation version 12 released 10 July, 2017, Reference List *Homo sapiens*). We excluded metabolic pathways for the heat map presentation of enriched pathways. A full list of results is provided in Suppl. Table 3.

### Comparison to non-IEM diseases

To perform a network-based comparison to other disease classes we used a previously described measure (Menche et al 2015). The separation score compares the mean shortest distance of protein pairs of the same disease to the mean shortest distance between protein pairs of different diseases. We calculated the separation score for every pair of a IEM disease group to a non-IEM disease. For non-IEM diseases we used a set of 299 diseases based on MeSH terms and compiled by Menche et al.

### Drug target information

We downloaded drug target information from the DrugBank database as of July 16, 2017 (https://www.drugbank.ca, DrugBank Release Version 5.0.7). In more detail, we used the *approved Target Drug-UniProt* sheet to retrieve only approved drug target information for our proteins. We mapped UniProt IDs to Entrez Gene IDs and found 634 approved drugs including small molecules and biotech drugs targeting 316 non-IEM binding proteins.

### Software

We used Cytoscape V.3.5.1 for the drawing and coloring of all networks. Network layouts were obtained using the built-in layout algorithm *organic*. The network layout for Fig. 3e was done manually. Basic network measures were calculated using the Cytoscape plugin *NetworkAnalyzer*.

## Electronic supplementary material

Suppl. Table 1 (XLSX 63 kb)

Suppl. Table 2 (XLSX 156 kb)

Suppl. Table 3 (XLSX 18 kb)

Suppl. Table 4 (XLSX 52 kb)

Suppl. Table 5 (XLSX 46 kb)

Suppl. Fig. 1 (GIF 137 kb)

High resolution image (TIFF 1324 kb)
