# Supplementary figures and images for: Inborn errors of metabolism and the human interactome: a systems medicine approach
Source: J Inherit Metab Dis. 2018 Feb 5;41(3):285–96. doi: 10.1007/s10545-018-0140-0 (PMC5959957; doi:10.1007/s10545-018-0140-0)

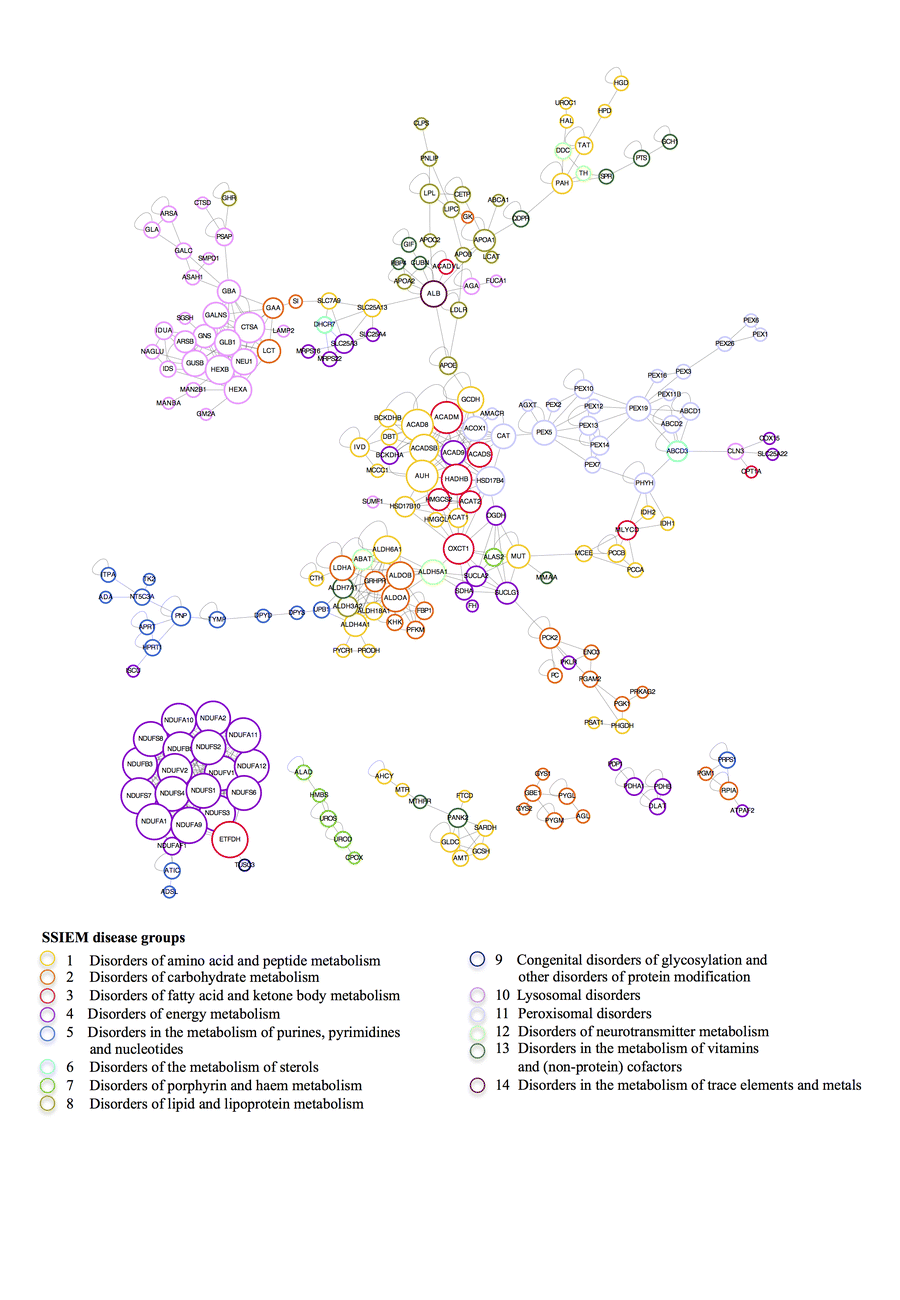

Supplement: Supplementary file 6 — (GIF 137 kb) [file 10545_2018_140_Fig5_ESM.gif]

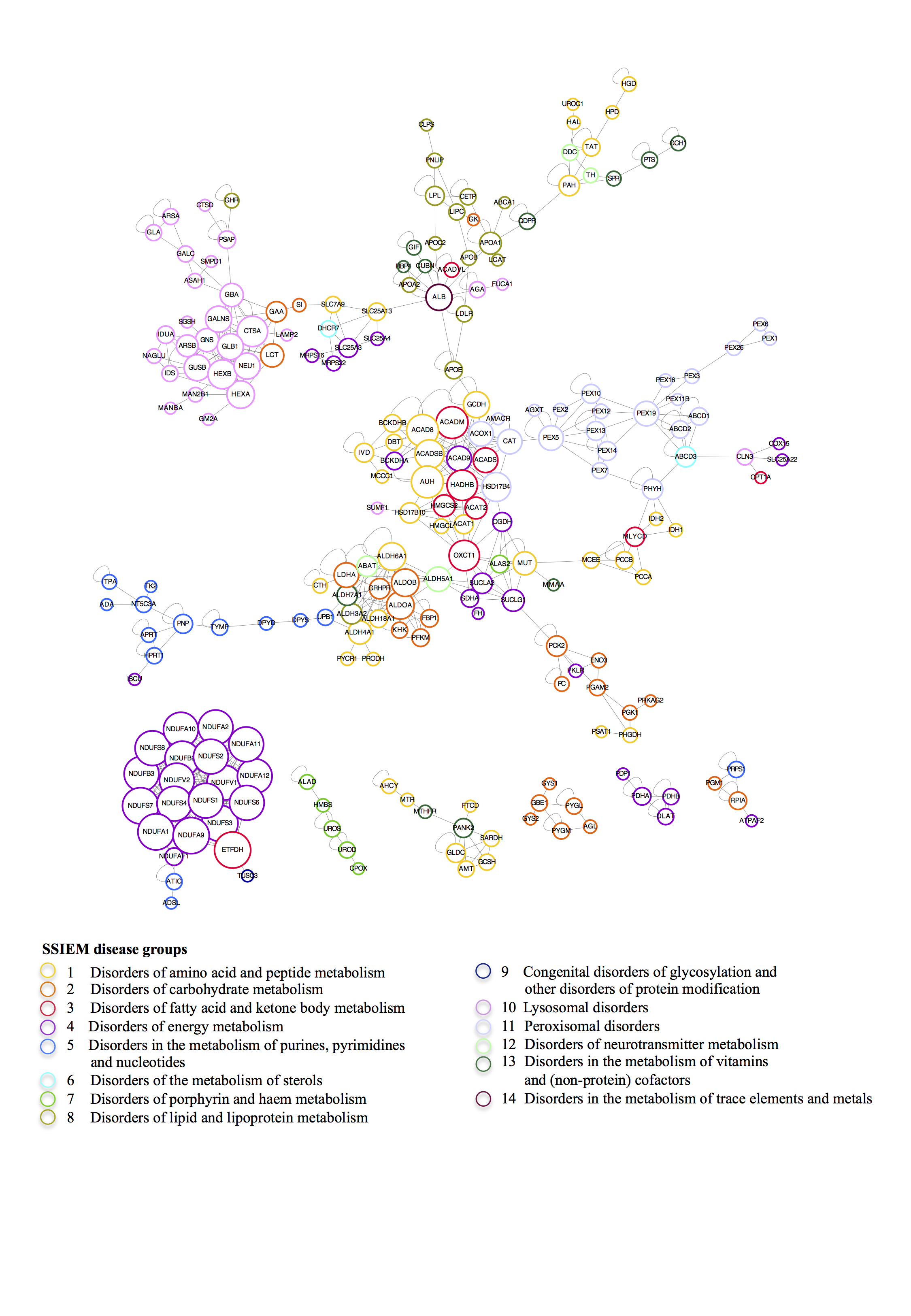

Supplement: Supplementary file 7 — High resolution image (TIFF 1324 kb) [file 10545_2018_140_MOESM6_ESM.tiff]
